# Narrow QRS Tachycardia with Alternate Wide QRS Beats: What is the Mechanism?

**Published:** 2008-08-01

**Authors:** Kartikeya Bhargava, Rajesh Jindal, Tarlochan S Kler

**Affiliations:** Department of Cardiac Arrhythmia Services, Escorts Heart Institute and Research Centre, New Delhi, India

**Keywords:** atrioventricular reciprocating tachycardia, preexcitation, bigeminy

## Case Presentation

**Figure 1:**
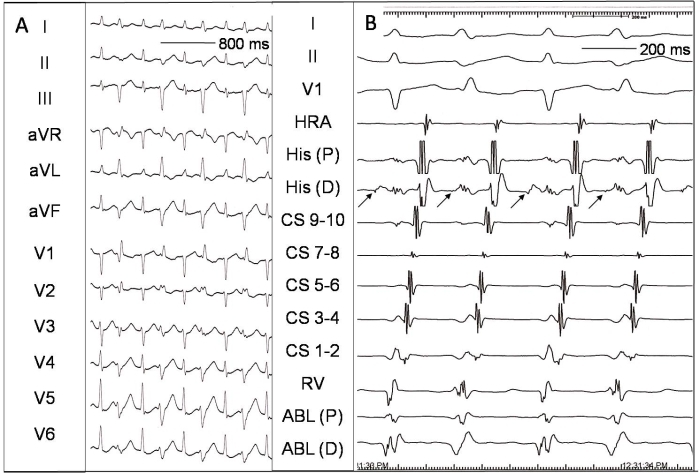
***Panel A.*** Twelve lead ECG (at 25 mm/sec) during orthodromic atrioventricular reciprocating tachycardia showing alternate wide QRS beats of RBBB morphology and left axis deviation. ***Panel B.***Intracardiac electrograms (at 100 mm/sec) during the same time as in panel A. Note that the wide QRS complexes are premature and are preceded by shorter AH interval and very short HV interval. The earliest ventricular activation during wide QRS beats is seen in the distal ablation recording. The arrows depict His bundle deflection. From top to bottom are surface ECG leads I, II and V1, high right atrium (HRA), His bundle proximal [His (P)], His bundle distal [His (D)], coronary sinus proximal (CS 9-10) to distal (CS 1-2), right ventricle (RV) and ablation proximal [ABL(P)] and distal [ABL(D)] recordings.

A 44-year old lady underwent electrophysiology study for recurrent palpitations and documented narrow QRS regular tachycardia. The baseline ECG showed subtle preexcitation that was easily manifest on atrial pacing. The retrograde atrial activation sequence during ventricular pacing was eccentric suggesting retrograde conduction over the accessory pathway. A regular narrow QRS tachycardia with cycle length 280 ms was easily inducible on programmed atrial stimulation. The earliest ventricular activation during sinus rhythm and atrial pacing, and the retrograde atrial activation during ventricular pacing and tachycardia were diagnostic of left free wall accessory pathway and orthodromic atrioventricular reciprocating tachycardia (AVRT). During an episode of tachycardia, an ablation catheter was placed in the region of the lateral mitral annulus using retrograde trans-aortic approach.  Once the ablation catheter was stabilized in that region, an interesting change in the AVRT was seen with appearance of wide QRS complexes of right bundle branch block (RBBB) morphology and left axis deviation in the alternate beats (Figure 1). The atrial activation sequence during both narrow and wide QRS beats was same with earliest activation in the distal coronary sinus. What is the mechanism of the alternate wide QRS beats during the AVRT?

## Discussion

**Figure 2:**
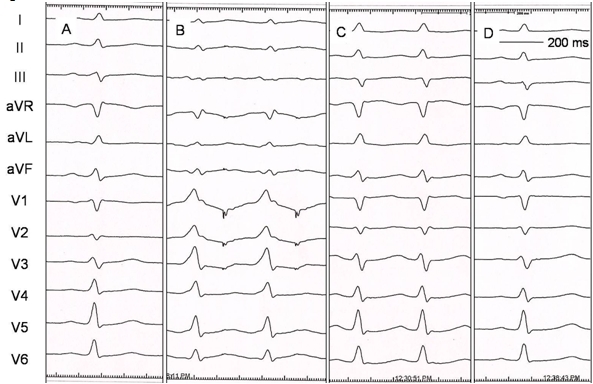
Twelve lead ECG recording during sinus rhythm (A), atrial pacing (B), orthodromic reciprocating tachycardia (C) and post radiofrequency ablation of accessory pathway (D).

**Figure 3:**
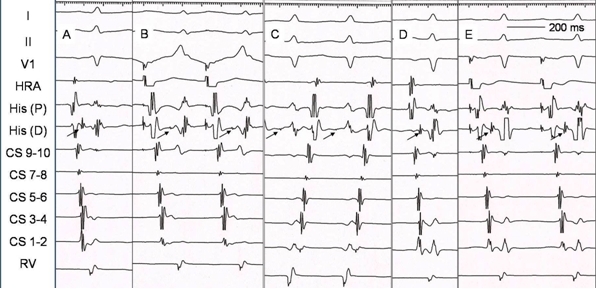
Intracardiac electrograms during sinus rhythm at baseline (A), atrial pacing (B), orthodromic reciprocating tachycardia (C), sinus rhythm after ablation (D) and atrial pacing after ablation (E). Note that though there is subtle preexcitation during sinus rhythm at baseline, the atrial and ventricular electrograms are nearly fused in the distal coronary sinus (A and B) and separate out after ablation (D and E). The arrows depict the His bundle deflection. From top to bottom are surface ECG leads I, II and V1, high right atrium (HRA), His bundle proximal [His (P)], His bundle distal [His (D)], coronary sinus proximal (CS 9-10) to distal (CS 1-2), and right ventricle (RV) recordings.

The baseline ECG in the present case showed subtle preexcitation (Figure 2A) that became prominent on atrial pacing (Figure 2B). The intracardiac electrograms during sinus rhythm (Figure 3B) and atrial pacing (Figure 3B) showed earliest ventricular activation in distal coronary sinus (CS 1-2) confirming the presence of antegradely conducting left free wall accessory pathway.  The narrow QRS tachycardia (Figure 2C) and its intracardiac electrograms (Figure 3C) showing earliest retrograde atrial activation in the distal coronary sinus were also suggestive of orthodromic AVRT, a diagnosis that was confirmed by pacing maneuvers. Thus, the patient had WPW syndrome with left free wall accessory pathway (with a match of atrial and ventricular insertion sites) and orthodromic AVRT.

Now, a careful look at Figure 1 reveals that the wide QRS beats that occur alternately are premature. The widening of premature QRS complexes in an otherwise narrow QRS tachycardia can be due to either aberrant intra-ventricular conduction, conduction over a bystander accessory pathway or ventricular premature beats (VPBs). The AH interval preceding the wide QRS beats is shorter (115 ms) compared to that preceding the narrow QRS (140 ms). Though, this may favor aberrant conduction, the important finding that HV interval during the wide QRS is only 10 ms or lesser excludes this possibility. A short HV interval indicates either preexcitation or VPB [1]. The earliest ventricular activity during the wide QRS beats is recorded at the distal ablation catheter that was lying at the ventricular aspect of the lateral mitral annular region. The intracardiac electrograms indicate that the retrograde atrial activation sequence in both the narrow and wide QRS complexes is identical with earliest activation in the distal coronary sinus. The ventriculo-atrial interval is also similar implying retrograde conduction over a single left free wall accessory pathway. Since the left free wall accessory pathway is conducting retrogradely, it cannot conduct antegradely at the same time. Hence, for the alternate wide QRS beats during AVRT to be preexcited, an additional left sided accessory pathway showing bystander antegrade conduction needs to be present. The additional pathway could also be a slowly conducting or decremental (Mahaim like) left sided accessory pathway [2]. However, absence of preexcitation during sinus rhythm and atrial pacing after ablation of the left free wall accessory pathway (see further below) excludes preexcitation as a cause of wide QRS beats. Thus, wide QRS complexes were due to VPBs occurring in a bigeminal fashion. The RBBB morphology with left axis in the frontal plane of the wide QRS complexes suggests origin near the left posterior fascicle. However, earliest ventricular activity in the distal ablation catheter indicates that the VPB's were arising from the lateral left ventricle (possibly induced by the ablation catheter trauma) and leftward axis was due to fusion of ventricular activation from antegrade conduction through His-purkinje system. Also, subtle differences in the QRS morphology during the wide QRS beats (see lead V1 in Figure 1A) indicate different degrees of fusion.

The accessory pathway was mapped by moving the ablation catheter slightly in the lateral mitral annulus to record the earliest retrograde atrial activation during the tachycardia. Radiofrequency application at this site promptly terminated the tachycardia with loss of preexcitation during sinus rhythm (Figure 2D and 3D). Atrial pacing after ablation showed absence of ventricular preexcitation (Figure 3E). Right ventricular pacing showed concentric retrograde atrial activation and no tachycardia could be induced by either atrial or ventricular stimulation. Adenosine injection resulted in atrioventricular block during sinus rhythm and ventriculo-atrial block during ventricular pacing.

Thus, the tracing in Figure 1 is orthodromic AVRT due to left free wall accessory pathway with ventricular bigeminy due to catheter induced VPBs. The interesting feature was that the VPBs were induced only during the alternate beats and that could be related to the effects of cardiac motion during the tachycardia.
